# A fast regularized least-squares method for retinal vascular oxygen tension estimation using a phosphorescence lifetime imaging model

**DOI:** 10.1186/1475-925X-12-106

**Published:** 2013-10-16

**Authors:** Gokhan Gunay, Isa Yildirim

**Affiliations:** 1 Electrical and Electronics Engineering Department, Istanbul Technical University, 34469 Istanbul, Turkey; 2 College of Engineering Department, University of Illinois at Chicago, 60607 Chicago, IL, USA

**Keywords:** Accelerated estimation, Closed-form solution, Regularized estimation, Retinal oxygen tension, Phosphorescence lifetime imaging

## Abstract

**Background:**

Monitoring retinal oxygenation is of primary importance in detecting the presence of some common eye diseases. To improve the estimation of oxygen tension in retinal vessels, regularized least-squares (RLS) method was shown to be very effective compared with the conventional least-squares (LS) estimation. In this study, we propose an accelerated RLS estimation method for the problem of assessing the oxygenation of retinal vessels from phosphorescence lifetime images.

**Methods:**

In the previous work, gradient descent algorithms were used to find the minimum of the RLS cost function. This approach is computationally expensive, especially when the oxygen tension map is large. In this study, using a closed-form solution of the RLS estimation and some inherent properties of the problem at hand, the RLS process is reduced to the weighted averaging of the LS estimates. This decreases the computational complexity of the RLS estimation considerably without sacrificing its performance.

**Results:**

Performance analyses are conducted using both real and simulated data sets. In terms of computational complexity, the proposed RLS estimation method is significantly better than RLS methods that use gradient descent algorithms to find the minimum of the cost function. Additionally, there is no significant difference between the estimates acquired by the proposed and conventional RLS estimation methods.

**Conclusion:**

The proposed RLS estimation method for computing the retinal oxygen tension is computationally efficient, and produces estimates with negligible difference from those obtained by iterative RLS methods. Further, the results of this study can be applied to other lifetime imaging problems that have similar properties.

## Background

Retinal tissue requires regular oxygenation to prevent some devastating eye diseases, such as diabetic retinopathy, glaucoma, and age-related macular degeneration [1,2]. Oxygen tension in retinal vessels should therefore be monitored for use in the early diagnosis of these devastating eye diseases.

Oxygen sensitive microelectrodes can be used to obtain retinal oxygenation [3]. However, this is an invasive method, as the electrodes damage the microenvironment of the retina. Magnetic resonance imaging, a noninvasive imaging technique, has been used to measure retinal oxygen tension [4]. This approach is limited by its low resolution compared with optical imaging methods.

The oxygen tension of retinal vessels can also be measured using a phosphorescence lifetime imaging model (PLIM) [5-7]. PLIM is a noninvasive method, though it requires intravenous injection, and does not damage the microenvironment of the retina. The least-squares (LS) estimation method has been used to estimate the oxygenation of retinal vessels based on PLIM. However, the LS method is not robust against noise contamination, producing high variance and artificial peaks in the estimates, as well as values outside of the physiological range, which can be attributed to the no use of a suitable prior model for the data.

To overcome these shortcomings of the LS method, a regularized LS (RLS) estimation method was proposed [8]. The RLS method was developed by utilizing knowledge of the prior distribution of the model parameters. By applying the RLS method to simulated data as well as real image data acquired from rat retinas, the method was shown to be robust to the presence of noise, and generated estimates that were more in the physiologically expected range and whose variance was lower than that obtained with the LS method which does not use a suitable prior model for the data.

Successful applications of regularization in image processing [9], biomedical engineering and imaging [10-13], and astronomical imaging [14] motivated the development of an RLS estimation method for oxygen tension in retinal blood vessels using PLIM [8]. In [8], the regularization window was chosen as a 3×3 window, equivalent to 15x15 *μm*^2^. The choice of this size of the window was inspired by the fact that variation among oxygen tension values in a neighborhood of this size of window should physiologically be negligible [15]. The regularization term in the RLS cost function was developed using this physiological information and assuming that the mean of a pixel in an oxygen tension map of retinal blood vessels is equal to the sample mean of oxygen tension values in neighboring pixels. The RLS estimation method was shown to be superior to the conventional LS estimation method as mentioned before. However, the gradient-based iterative methods used to find the minimum of the RLS cost function are computationally costly, especially when considering large oxygen tension maps.

Existing applications have a typical oxygen tension map frame size of around 512 × 160 pixels. In the early diagnosis of some eye diseases, analyses of small capillaries are of great importance and require imaging systems with higher resolutions and larger frame sizes. However, as the map frame size becomes larger, the computational cost of implementing the RLS estimation method increases drastically. Therefore, to realize RLS estimation for large oxygen tension maps, faster estimation methods are needed.

In the literature, fast regularized estimation methods have been proposed. For instance, Toh and Yun proposed an accelerated proximal gradient method to minimize a non-smooth convex regularized cost function [16], and Lampe and Voss proposed a fast algorithm for Tikhonov-based regularized total LS estimation problems [17]. Mastronardi, Lemmerling and Van Huffel provided a fast regularized total LS algorithm for solving the basic deconvolution problem [18].

In this study, we propose a computationally efficient RLS method for estimating retinal oxygen tension using a closed-form solution. The proposed method is shown to be much faster than the iterative solution, and generates almost identical results. Additionally, the approach derived in this study can be applied to other imaging problems using PLIM or FLIM with some minor changes.

## Past RLS estimation method

### LS estimation of retinal oxygen tension

The Stern–Volmer equation, giving the relationship between the oxygen tension *pO*_2_ and the phosphorescence lifetime *τ*, is as follows:

(1) $$\frac{\tau_{0}}{\tau }=1+K_{\varphi }\tau_{0}\left(pO_{2}\right),$$

where *pO*_2_ (in millimeters of mercury) denotes oxygen tension, K_*ϕ*_ is the quenching constant, and τ_0_ denotes the lifetime in a zero oxygen environment. Based on Eq. (1), we need to estimate *τ* to find the value of *pO*_2_. The phosphorescence lifetime τ can be calculated from the distribution of phosphorescence intensity values in different modulation phase images, because the intensity depends on the phase angle, denoted by *θ*, between the modulated excitation laser light and the emitted phosphorescence [5,6]. The relation between *θ* and τ is

(2) tanθ=ωτ,

where *ω* is the modulation frequency. The intensity can be represented as a function of *θ*_*n*_,

(3) $$\begin{array}{ll} I\left(\mathrm{\theta n}\right)= & k\left[\mathrm{Pd}\right]\\ & +\frac{1}{2}k\left[\mathrm{Pd}\right]mm_{n}\left(\cos\left(\theta \right)\cos\left(\mathrm{\theta n}\right)+\sin\left(\theta \right)\sin\left(\mathrm{\theta n}\right)\right)\cdots \cdots n=1,2,\;\ldots ,n_{p} \end{array}$$

where m_n_, *θ*_*n*_, and *n*_*p*_ denote the modulation profile of the image intensifier, the phase of the gain modulation, and the number of phosphorescence intensity observations at each pixel location for different phase values of *θ*_*n*_, respectively. The concentration of the probe [*Pd*], *k*, and the modulation *m* are unavailable.

If we define *a*_0_ = *k*[Pd], *a*_1_ = (1/2)*k*[Pd]*mm*_*n*_cos(*θ*), and *b*_1_ = (1/2)*k*[Pd]*mm*_*n*_sin(*θ*), we can express Eq. (3) as:

(4) $$I\left(\mathrm{\theta n}\right)=a_{0}+a_{1}\cos\left(\mathrm{\theta n}\right)+b_{1}\sin\left(\mathrm{\theta n}\right).$$

The phase angle *θ* in Eq. (3) is obtained as:

(5) $$\theta =\mathrm{ta}n^{-1}\left(b_{1}/a_{1}\right).$$

Therefore, using Equations (1), (2), and (5), we are able to acquire values for *pO*_2_ at each observation location. Here it should be stressed that *pO*_2_ in the Equation 1 can also be found using phosphorescence quenching measurement. However, for multiple and closely spaced lifetime applications, the PLIM method satisfies needs of the application more [19].

In the presence of additive noise and for multiple observations, Eq. (4) can be written in matrix–vector form as:

(6) $$\left[\begin{array}{c} I_{1}^{i}\\ I_{2}^{i}\\ \vdots \\ \vdots \\ I_{n}^{i} \end{array}\right]=\left[\begin{array}{c} 1\;\cos\left(\theta_{1}\right)\;\sin(\theta_{1})\\ 1\;\cos\left(\theta_{2}\right)\;\sin(\theta_{2})\\ \vdots \\ \vdots \\ 1\;\cos\left(\theta_{n}\right)\;\sin(\theta_{n}) \end{array},\;\right]\;\left[\begin{array}{l} a_{0}^{i}\\ a_{1}^{i}\\ b_{1}^{i} \end{array}\right]\;+\;\left[\begin{array}{c} N_{1}^{i}\\ N_{2}^{i}\\ \vdots \\ \vdots \\ N_{n}^{i} \end{array}\right]$$

where *i* denotes the *i-th* pixel and *n* denotes the number of phosphorescence intensity observations for each pixel. We can rewrite Eq. (6) as follows:

(7) y=Ax+n

where ***y***, ***A***, ***x***, and ***n*** are the phosphorescence lifetime intensity observations, system matrix, PLIM parameters, and additive noise, respectively. Clearly, to find the model parameters *a*_*0*_, *a*_*1*_, and *b*_*1*_, at least three phosphorescence intensity observations must be obtained at three different gain modulation phases for each pixel, though in practice we need more than three observations because of noise contamination. The LS estimate of the model parameters is obtained as follows:

(8) $${\left[{\hat{a}}_{0}\;{\hat{a}}_{1}\;{\hat{b}}_{1}\right]}^{T}=Q{\underline{I}}^{i},$$

where ***Q*** is the pseudo-inverse of the system matrix in Eq. (7).

### RLS estimation of retinal oxygen tension

In [14], the RLS cost function for the parameter *a*_1_ was defined as:

(9) $$f_{a_{1}}^{i}={\left(a_{1}^{i}-Q\left(2,:\right)y^{i}\right)}^{2}+\beta {\left(a_{1}^{i}-{\bar{a}}_{1}^{i}\right)}^{2}$$

where ***Q*** (2,:) ***y***^*i*^ is the LS estimate of the parameter $a_{1}^{i},$ ***Q*** (2,:) is the second row of the pseudo-inverse of the system matrix ***A*** in Eq. (7), ***y***^*i*^ is the phosphorescence intensity observation vector of the *i-th* pixel, and *β* is the regularization parameter. ${\bar{a}}_{1}^{i}$ denotes the mean value of the parameter to be estimated for the *i-th* pixel. The global cost function was defined as:

(10) $$F_{a_{1}}=\sum_{1}^{M}f_{a_{1}}^{i},$$

where *M* denotes the total number of pixels in the image. A gradient-based iterative approach was then used to find the minimum of the RLS cost function.

### Derivation of the proposed solution for the RLS estimation

The RLS cost function for the model parameters *a*_*0*_, *a*_*1*_, and *b*_*1*_ for the *i-th* pixel is defined as:

(11) $$C_{\mathrm{RLS}}^{i}=∥y^{i})+{(Ax^{i}∥}_{2}^{2}+(\gamma ∥x^{i}-{({\bar{x}}^{i}∥}_{2}^{2},$$

where ***y***^*i*^ is the noisy observation vector, ***A*** is the system matrix, and *γ* is a regularization coefficient. In Eq. (11), ***x***^*i*^ is the parameter vector and ${\bar{x}}^{i}$ is its sample mean, which are defined as:

(12) $$x^{i}={\left[a_{0}^{i}\;a_{1}^{i}\;b_{1}^{i}\right]}^{T}\;\mathrm{and}\;{\bar{x}}^{i}={\left[K\left(i,:\right)a_{0}\;K\left(i,:\right)a_{1}\;K\left(i,:\right)b_{1}\right]}^{T},$$

where ***a***_0_, ***a***_1_, and ***b***_1_ are vectors of the phosphorescence lifetime imaging model parameters and ***K*** is a weighting matrix defining the relations between these parameters. ***K*** is defined as follows:

(13) $$K\left(j,k\right)=\left\{\begin{array}{l} l\;\mathrm{if}\;\left|j-\left(k\right|=0\right)\\ p\;\mathrm{if}\;\left|j-\left(k\right|=1\;\mathrm{or}\;R\right)\\ q\;\mathrm{if}\;\left|j-\left(k\right|=R\pm 1\right)\\ 0\;\mathrm{otherwise} \end{array}\right)$$

where *l*, *p*, and *q* denote the weights of a pixel with respect to itself, to directly adjacent pixels, and to cross-adjacent pixels, respectively. These are chosen such that *l+4p+4q=1*. ***K*** *(j,k)* denotes the weight coefficient of the *k-th* pixel on the *j-th* pixel in the oxygen tension map, and ***R*** denotes the number of rows in the map. Note that ***K*** is a sparse positive-definite symmetric Toeplitz matrix.

From Equations (11) and (12), we see that the cost function for a pixel is dependent on the values of its neighboring pixels. Therefore, there is no pixel-wise solution, and the problem must be dealt with globally. The global cost function is written as:

(14) $$C_{\mathrm{RLS}}=\sum_{i=1}^{M}C_{\mathrm{RLS}}^{i},$$

where *M* is the number of pixels in the oxygen tension map.

For any **ℜ**^*IxJ*^, ǁ ***X*** ǁ_F_ is called the Frobenius norm in **ℜ**^*IxJ*^ space, and

(15) $$∥X{∥}_{F}=\sqrt{\mathrm{tr}\left(X^{T}X\right)}=\sqrt{\mathrm{tr}\left(XX^{T}\right)}=\sqrt{\sum_{i=1}^{I}\sum_{j=1}^{J}X_{\mathrm{ij}}X_{\mathrm{ij}},}$$

where *tr* denotes the trace of a matrix. The operation *tr*(***X***^*T*^ ***X***) is called the Frobenius inner product [20]. The global cost function using the Frobenius inner product can now be written as:

(16) $$C_{\mathrm{RLS}}=∥Y-\mathrm{AX}{∥}_{F}^{2}+\;\gamma ∥X-\bar{X}{∥}_{F}^{2}.$$

***X*** and ***Y*** in Eq. (16) are defined as:

(17) $$x=\left|\begin{array}{c} a_{0}^{1}\cdots a_{0}^{i}\cdots a_{0}^{M}\\ a_{1}^{1}\cdots a_{1}^{i}\cdots a_{1}^{M}\\ b_{1}^{1}\cdots b_{1}^{i}\cdots b_{1}^{M} \end{array}\right|=\left[x^{1}\ldots x^{i}\ldots x^{M}\right]\;\mathrm{and}\;Y=\left|\begin{array}{c} I_{1}^{1}\cdots I_{1}^{i}\cdots I_{1}^{M}\\ \vdots \cdots \vdots \;\cdots \vdots \\ I_{S}^{1}\cdots I_{S}^{i}\cdots I_{S}^{M} \end{array}\right|,$$

where $I_{s}^{i}$ and *S* are the *s-th* phosphorescence intensity observation of the *i-th* pixel and the number of observations per pixel, respectively. Given the definition of ***X***, Eqs. (12) and (16) can be rewritten as follows:

(18) $${\bar{x}}^{i}={\left(K\left(i,:\right)X^{T}\right)}^{T},$$

(19) $$C_{\mathrm{RLS}}=\;∥Y-\mathrm{AX}{∥}_{F}^{2}+\;\gamma ∥X-{\left(KX^{T}\right)}^{T}{∥}_{F}^{2}.$$

For the model parameters, the regularization term in the cost function (19) can be expanded as:

(20) $$∥X-{\left(KX^{T}\right)}^{T}{∥}_{F}^{2}=∥a_{0}-Ka_{0}{∥}_{2}^{2}+\;∥a_{1}-Ka_{1}{∥}_{2}^{2}+\;∥b_{1}-Kb_{1}{∥}_{2}^{2}.$$

The least-squares term in (19) can be rewritten as:

(21) $$∥Y-\mathrm{AX}{∥}_{F}^{2}=\mathrm{tr}\left(Y^{T}Y\right)-2\mathrm{tr}\left(Y^{T}\mathrm{AX}\right)+\mathrm{tr}\left(X^{T}A^{T}\mathrm{AX}\right).$$

Because ***A***^*T*^ ***A*** is equal to

(22) $$A^{T}A=\left|\begin{array}{c} S\;0\;0\\ 0\;S/2\;0\\ 0\;0\;S/2 \end{array}\right|,$$

*tr*(***X***^*T*^ ***A***^*T*^ ***AX***) becomes

(23) $$\mathrm{tr}\left(X^{T}A^{T}\mathrm{AX}\right)=Sa_{0}^{T}a_{0}+\frac{S}{2}a_{1}^{T}a_{1}+\frac{S}{2}b_{1}^{T}b_{1}.$$

From the definition of the Frobenius inner product, *tr*(***Y***^T^ ***AX***) can be rewritten as:

(24) $$\mathrm{tr}\left(Y^{T}\mathrm{AX}\right)=\mathrm{tr}\left(XY^{T}A\right)=a_{0}^{T}Y^{T}A\left(:,1\right)+a_{1}^{T}Y^{T}A\left(:,2\right)+b_{1}^{T}Y^{T}A\left(:,3\right).$$

Considering the above equations, the global cost function can now be written as:

(25) $$\begin{array}{l} C_{\mathrm{RLS}}=\mathrm{tr}\left(Y^{T}Y\right)+a_{0}^{T}\left(Sa_{0}-2Y^{T}A\left(:,1\right)\right)+\\ a_{1}^{T}\left(\frac{S}{2}a_{1}-2Y^{T}A\left(:,2\right)\right)+b_{1}^{T}\left(\frac{S}{2}b_{1}-2Y^{T}A\left(:,3\right)\right)+\\ \gamma \left(∥a_{0}-Ka_{0}{∥}_{2}^{2}+∥a_{1}-Ka_{1}{∥}_{2}^{2}+∥b_{1}-Kb_{1}{∥}_{2}^{2}\right). \end{array}$$

We need the model parameters ***a***_1_ and ***b***_1_ to estimate the oxygen tension, because this is nonlinearly dependent on the ratio of ***b***_1_ and ***a***_1_. Taking the gradient of the cost function with respect to the model parameters and equating to zero, we obtain the RLS estimates of the model parameters.

(26) $$\nabla_{a_{1}}C_{\mathrm{RLS}}=\left(Sa_{1}-2Y^{T}A\left(:,2\right)\right)+\mathrm{S\beta}\left(I-2K+K^{2}\right)a_{1}=0,$$

(27) $${\hat{a}}_{1-\mathrm{RLS}}={\left(I+\beta \left(I-2K+K^{2}\right)\right)}^{-1}\left(\frac{2}{S}Y^{T}A\left(:,2\right)\right),$$

(28) $$\nabla_{b_{1}}C_{\mathrm{RLS}}=\left(Sb_{1}-2Y^{T}A\left(:,3\right)\right)+\mathrm{S\beta}\left(I-2K+K^{2}\right)b_{1}=0,$$

(29) $${\hat{b}}_{1-\mathrm{RLS}}={\left(I+\beta \left(I-2K+K^{2}\right)\right)}^{-1}\left(\frac{2}{S}Y^{T}A\left(:,3\right)\right).$$

In Eqs. (27) and (29), $\left(\frac{2}{S}Y^{T}A\left(:,2\right)\right)\;\mathrm{and}\;\left(\frac{2}{S}Y^{T}A\left(:,3\right)\right)$ are the LS estimates of ***a***_1_ and ***b***_1_, respectively. Therefore, the RLS estimates of ***a***_1_ and ***b***_1_ can be written as:

(30) $${\hat{a}}_{1-\mathrm{RLS}}=(I+\beta {\left(I+K^{T}K-K-K^{T}\right)}^{-1}{\hat{a}}_{1},$$

(31) $${\hat{b}}_{1-\mathrm{RLS}}=(I+\beta {\left(I+K^{T}K-K-K^{T}\right)}^{-1}{\hat{b}}_{1},$$

where ${\hat{a}}_{1}$ and ${\hat{b}}_{1}$ are the LS estimates of these parameters. By defining a new matrix ***L*** as:

(32) $$L_{\mathrm{MxM}}=I+\beta \left(I+K^{T}K-K-K^{T}\right),$$

where ***I*** is the identity matrix, the RLS estimates of ***a***_1_ and ***b***_1_ can be rewritten in a simpler form as:

(33) $${\hat{a}}_{1-\mathrm{RLS}}=L^{-1}{\hat{a}}_{1},$$

(34) $${\hat{b}}_{1-\mathrm{RLS}}=L^{-1}{\hat{b}}_{1}.$$

The matrix ***K*** in Eq. (32) is a symmetric Toeplitz and positive-definite sparse matrix. Therefore, ***L*** is a symmetric positive-definite matrix. However, it is not in Toeplitz form because the matrix ***K***^*T*^ ***K*** is not in Toeplitz form. The elements of ***K***^*T*^ ***K*** can be written as:

(35) $${\left(K^{T}K\right)}_{\mathrm{ij}}=\sum_{k=1}^{M}K_{i\;k}K_{k\;j}=\sum_{k=1}^{M}K_{i\;k}K_{j\;k}.$$

Because *K*(*j,k*) = 0 *if* |*j* − *k*| > *R* + 1, all non-zero elements are within the range 2R+2, where R is the number of rows in the oxygen tension map. Thus, (***K***^*T*^ ***K***)_*i j*_ = 0 *if* |*i* −*j*| > 2*R* + 2.

If we ignore the first and last 2R+2 rows of the matrix ***K***^*T*^ ***K***, it is a positive-definite symmetric Toeplitz matrix. Additionally, as all matrices in ***L*** are in Toeplitz form except for ***K***^*T*^ ***K***, the matrix ***L*** has the same properties as ***K***^*T*^ ***K***. That is, except for the first and last 2R+2 rows, it is a purely positive-definite symmetric Toeplitz matrix. The inverse of ***L*** shares the same properties as ***L***. Therefore, if we choose a row between the first and last 2R+2 rows of the inverse of ***L***, we can reconstruct it with negligible errors in the first and last 2R+2 rows.

Further, even if we change the size of ***L***, there occurs only a negligible difference in the most significant elements of its inverse. This makes the matrix ***L*** and its inverse very useful when large *pO*_2_ images are being used. For a 640 × 480 *pO*_2_ map, ***L*** is a 307200 × 307200 matrix whose inverse cannot be calculated using a regular computer. In such cases, iterative approaches must be employed to find the optimum points of the RLS cost function, and these are also computationally expensive. Because the most significant elements in ***L***^**−1**^ do not vary considerably when its dimension is reduced, we can calculate a small $L_{s}^{-1}$ and acquire its most significant units, then extend the solution to larger *pO*_2_ maps with negligible errors.

**Figure 1:**
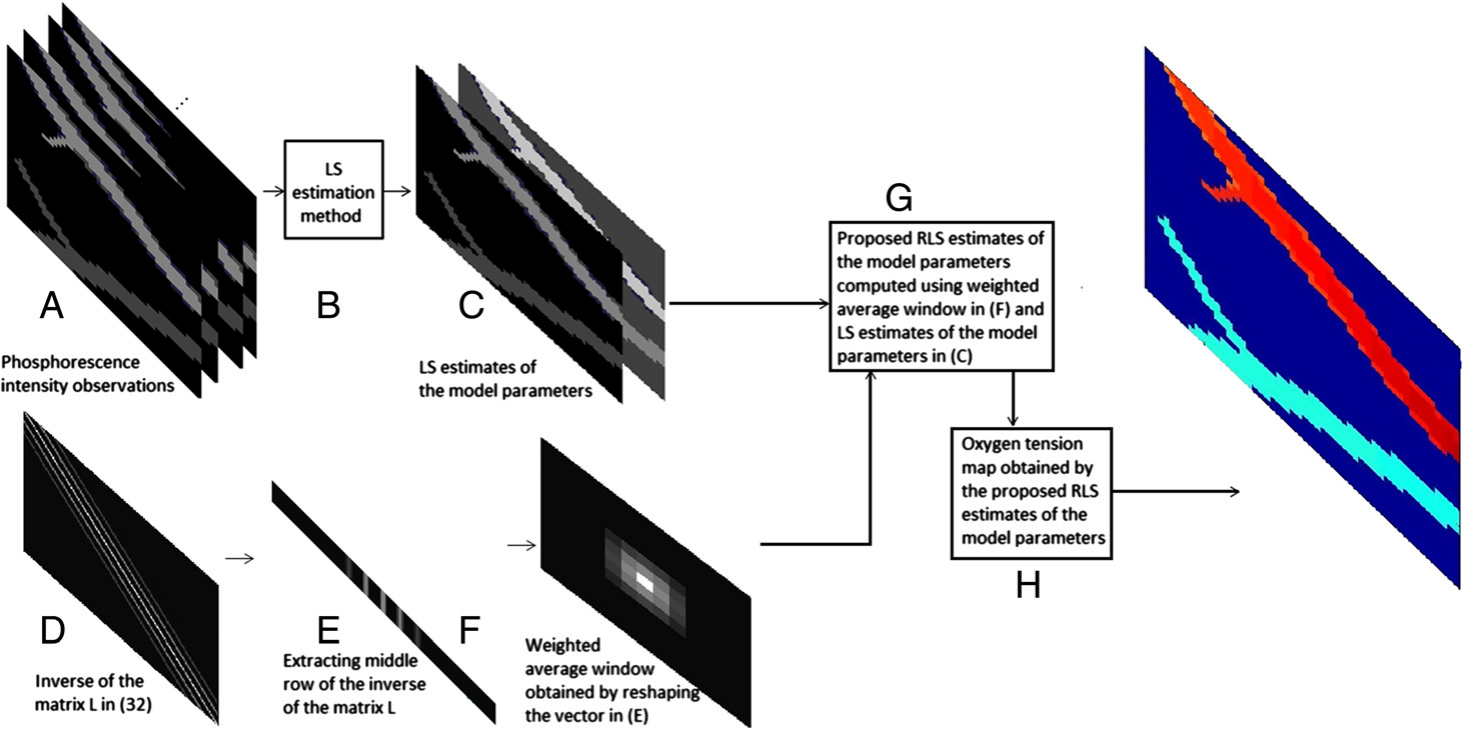
Flowchart of the proposed method.

The proposed method is visualized in Figure 1 as a flow chart: **(A)** Using the phosphorescence lifetime images, **(B-C)** the LS estimates of the model parameters *a*_1_ and *b*_1_ of the original *pO*_2_ map are found in matrix form. **(D)**$L_{s}^{-1}$, whose dimension is *R*_*s*_^2^*× R*_*s*_^2^, is found for a small *R*_*s*_*× R*_*s*_*pO*_2_ map, and **(E)** its middle row is chosen. *R*_*s*_ is chosen as an odd number to ensure symmetry with respect to the middle unit in the chosen row. Because $L_{s}^{-1}$ is in Toeplitz form except for the first and last two rows, all other rows can be recovered exactly using this row with appropriate shifts. Additionally, elements in the middle row that determine the weights to obtain approximate RLS estimates of the model parameters **(F)** can be reshaped to give an *R*_*s*_*× R*_*s*_ weighted averaging window. **(G)** Finally, this weighted averaging window is applied to the LS estimates of the *a*_1_ and *b*_1_ parameter maps to find approximate RLS estimates of the model parameters.

### Simulated and experimental data results

In the RLS method, the standard Newton–Raphson method is employed to find the minimum of the RLS cost function. The LS estimates are used as the initial values of the model parameters to be estimated. In the proposed RLS method, we first determine $L_{s}^{-1}$ as a 169 × 169 matrix for a 13 × 13 *pO*_2_ map. We then follow the procedure given in the previous section. The computer system used for these simulations is a standard notebook with an Intel Core Duo 2.27 GHz processor and 4 GB memory. Unless otherwise stated, the pre-set values of *β*, window size, and signal-to-noise ratio (SNR) are 5, 13 × 13, and 20 dB, respectively.

**Figure 2:**
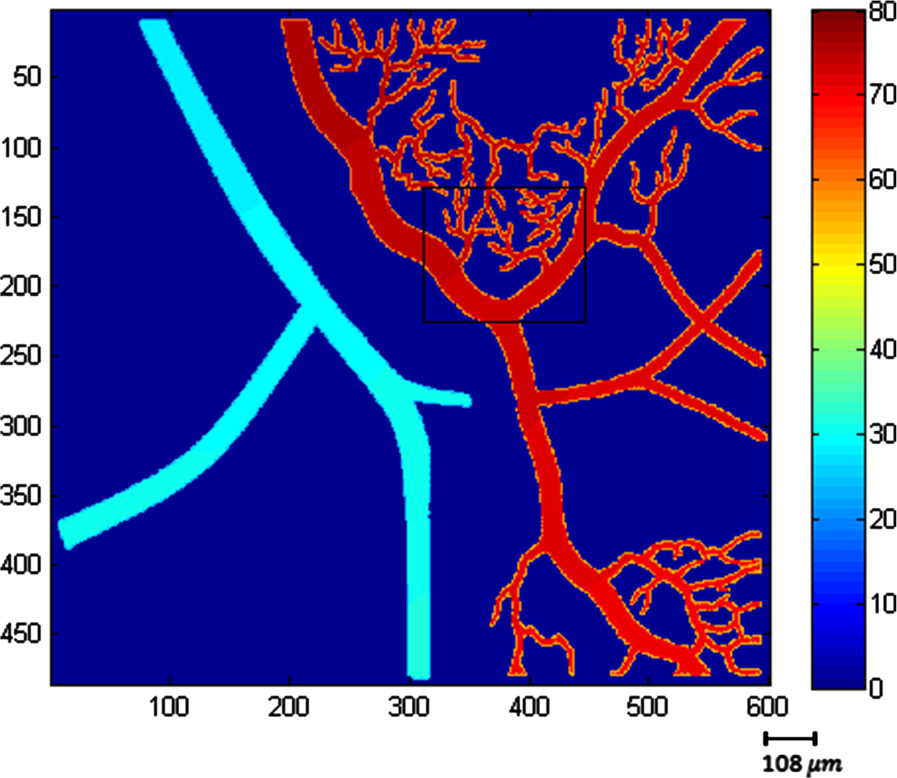
**Simulated *pO***_**2 **_**map (485 × 600 pixels).** Color bar represents *pO*_2_ values in millimeters of mercury.

**Figure 3:**
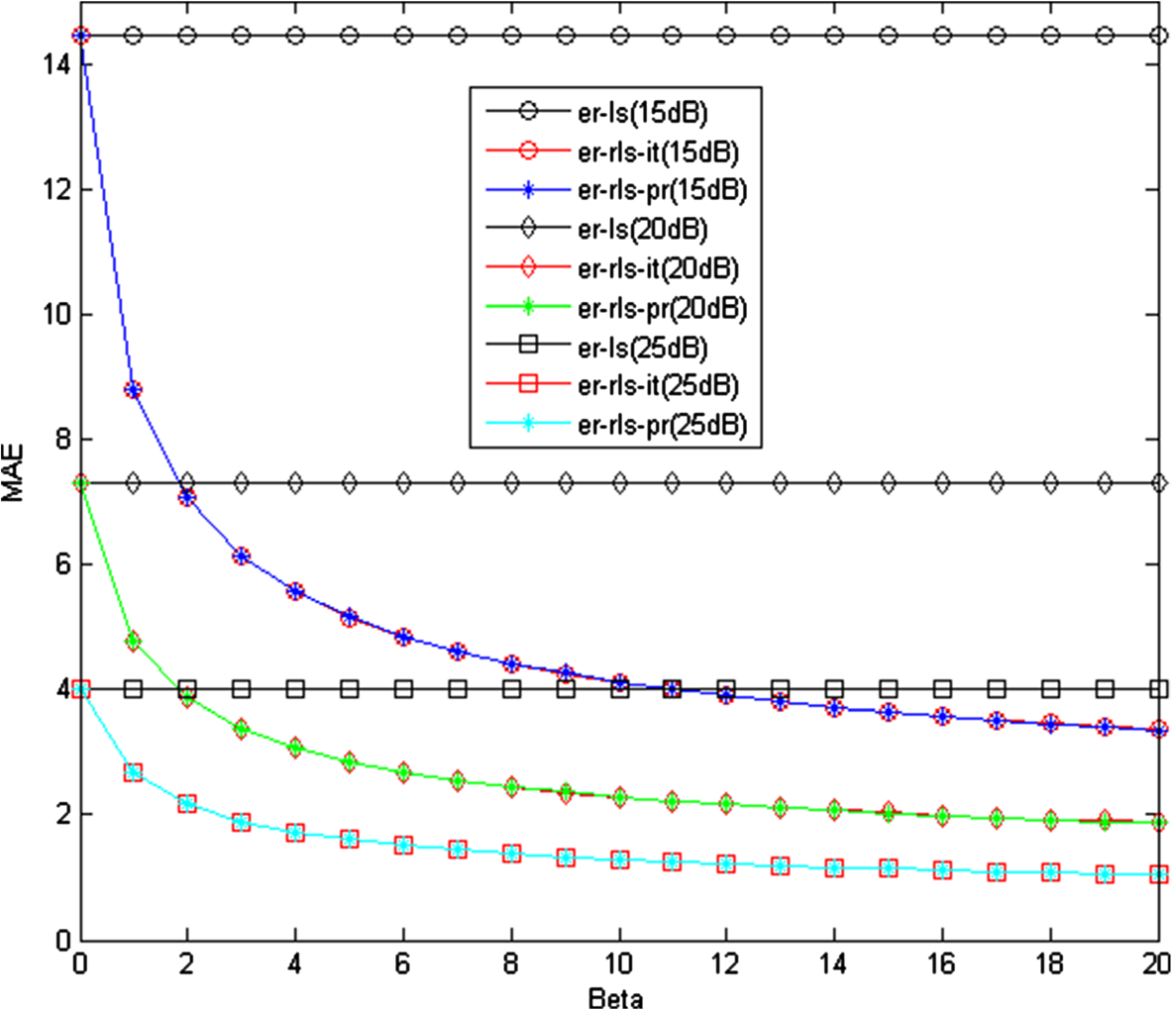
**Oxygen tension map (600 × 425 pixels) of a rat generated using the LS (1), iterative RLS (2), and the proposed RLS (3) methods.** The color bar shows oxygen tension in millimeters of mercury.

We use both simulated and real oxygen tension maps (Figures 2 and 3, respectively) in our experiments. The real retinal oxygen tension map used in this study was acquired from a Long Evans pigmented rat (~500 g) using a novel system described in [6]. The animal was treated according to the ARVO Statement for the Use of Animals in Ophthalmic and Vision Research. In order to anesthetize the rat, an intra-peritoneal infusion of Ketamine (85 mg/kg IP) and Xylazine (3.5 mg/kg IP) at the respective rates of 0.5 mg/kg/min and 0.02 mg/kg/min was used. Before and during the imaging, gas mixture containing 21% oxygen (room air, normoxia) was administered to rats for 5 minutes. Pd-porphine (Frontier Scientific, Logan, Utah), an oxygen-sensitive molecular probe, was dissolved (12 mg/ml) in bovine serum albumin solution (60 mg/ml) and physiological saline buffered to a pH of 7, and injected intravenously (20 mg/kg). Imaging was conducted at areas within two-disk diameters (600 microns) from the edge of the optic nerve head. For each pixel location, 10 phase-delayed optical section phosphorescence lifetime intensity images were acquired with a corresponding phase delay increments of 74 μs, and then these intensity images were brought together to form en-face phosphorescence images of the retinal vasculatures in the retinal plane. To generate the simulated data we used in this work, physiological features and topology of real retinal vessels were followed based on the previous studies [6-8,15]. Near the optic disc, which has a diameter nearly 300 microns [21], venous and arterial oxygen tensions of rat retina vary around 35 mm-Hg and 60 mm-Hg, respectively in the normoxia condition. As getting far from the optic disc, arterial oxygen tension decreases almost linearly while venous oxygen tension remains almost the same [15]. Since the iterative method requires a considerable amount of time as size of the *pO*_2_ map gets larger, the simulated *pO*_2_ map was generated to be 485x600 pixels. But it should be noted that this size is not a limitation to the proposed method. In the simulations, we add i.i.d. white Gaussian noise with 15, 20, and 25 dB SNR to the phosphorescence lifetime images.

**Figure 4:**
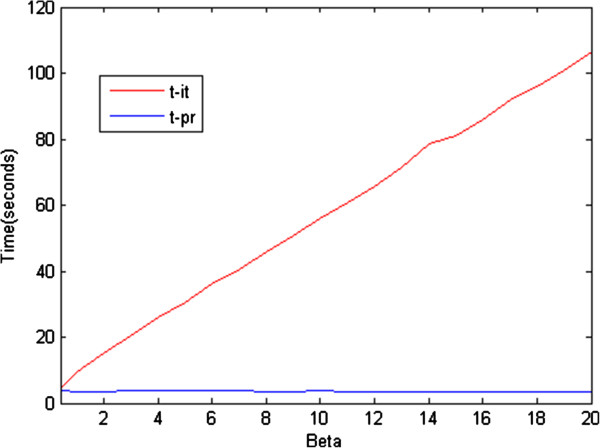
MAE of the LS (er-ls), iterative RLS (er-rls-it), and proposed RLS estimation (er-rls-pr) methods.

**Figure 5:**
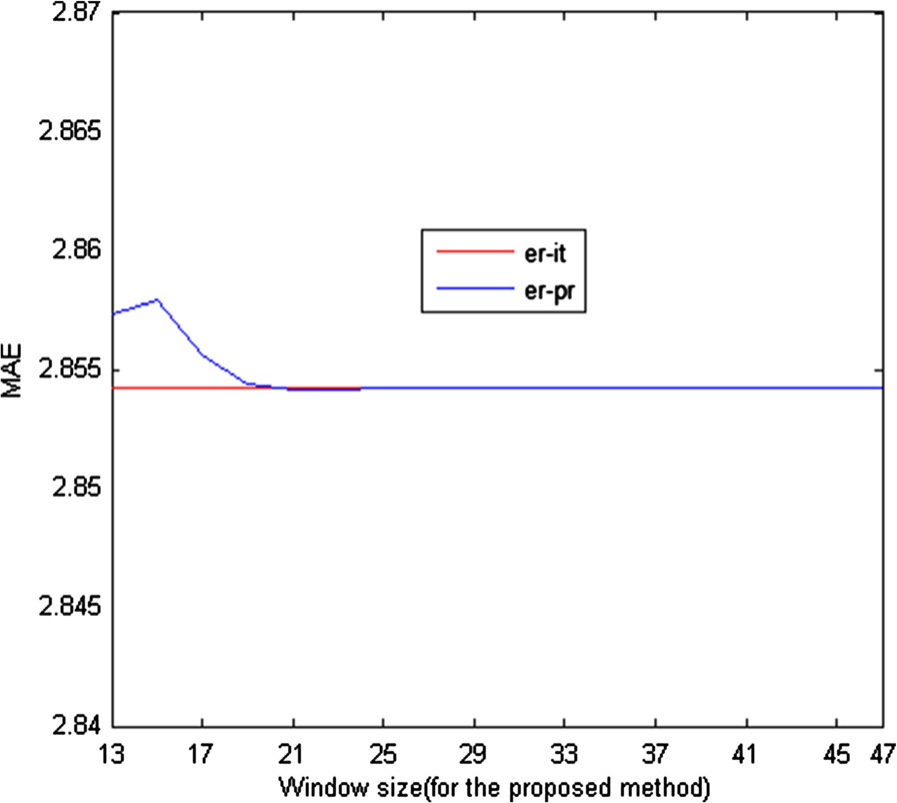
**Computation times of the iterative RLS (t-it) and proposed RLS (t-pr) estimation methods for different values of *β.***

We examined the MAE performance of the LS, iterative RLS, and proposed RLS methods for different regularization coefficient values. As shown in Figure 4, there is a negligible difference between the MAE of the iterative and proposed RLS estimation methods. On the other hand, there is a significant difference in computation time for the iterative and proposed RLS methods (see Figure 5). As mentioned previously, the Newton–Raphson method is used to minimize the RLS cost functions, and its step size, for our problem, is as follows:

(36) $$\delta ={\left(2\;+\;2\beta \left(l+4p+4q\right)\right)}^{-1}$$

Thus, the step size decreases for higher values of *β*, and this results in a proportional increase in computation time.

The time difference between the two methods would be much more notable if the *pO*_2_ map to be estimated were of higher dimensions. Therefore, it is clear that the proposed method is preferable to the iterative one in the sense of MAE performance versus computational complexity.

**Figure 6:**
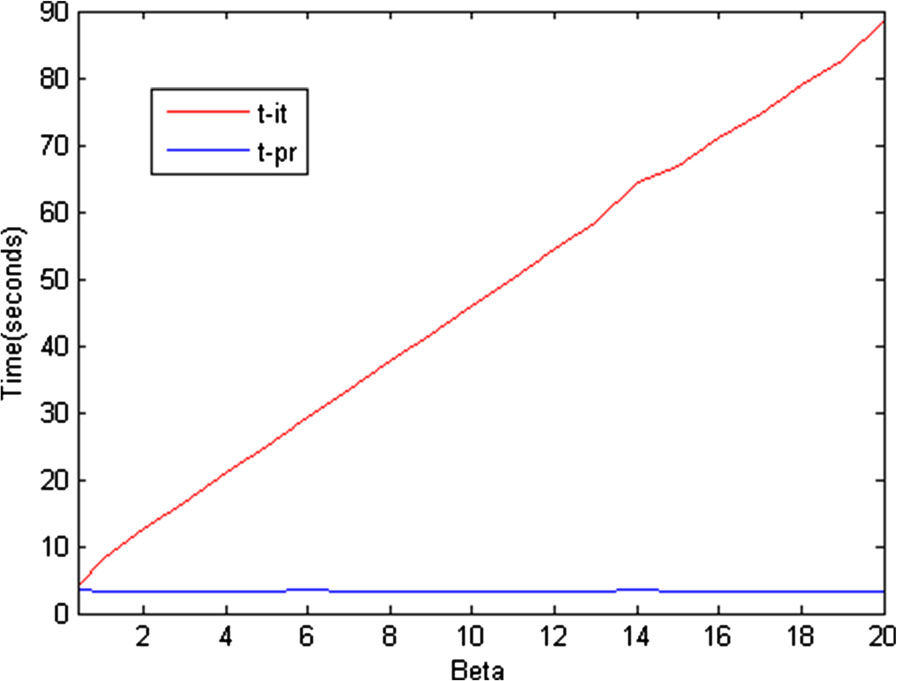
**MAE of the iterative RLS (er-it), and proposed RLS (er-pr) methods for different sizes of *pO***_**2 **_**maps used in the estimation of**$L_{s}^{-1}$**.**

From Figure 6, we can see that there is a small difference between the proposed and iterative approaches in terms of MAE performance for different sizes of the small *pO*_2_ maps used in the estimation of $L_{s}^{-1}$. As their size increases, the MAE of the proposed method converges to that of the standard iterative RLS method.

**Figure 7:**
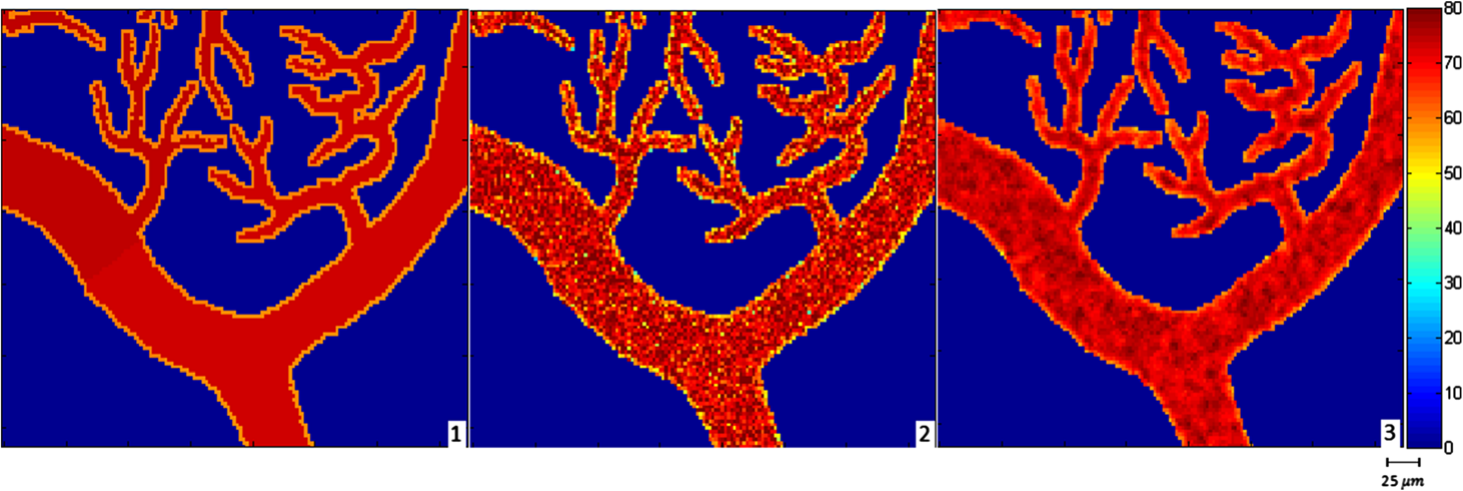
**Computation times of iterative RLS and proposed RLS estimation methods for different values of *β *** **for a real oxygen tension map.**

In Figure 7, we present the computation times of the iterative and proposed RLS estimation methods for a real oxygen tension map which was extended from original form 120x85 pixels to 600x425 pixels. As seen from Figures 4 and 6, the results for the real and simulated oxygen tension maps are proportional in terms of computation time.

**Figure 8:**
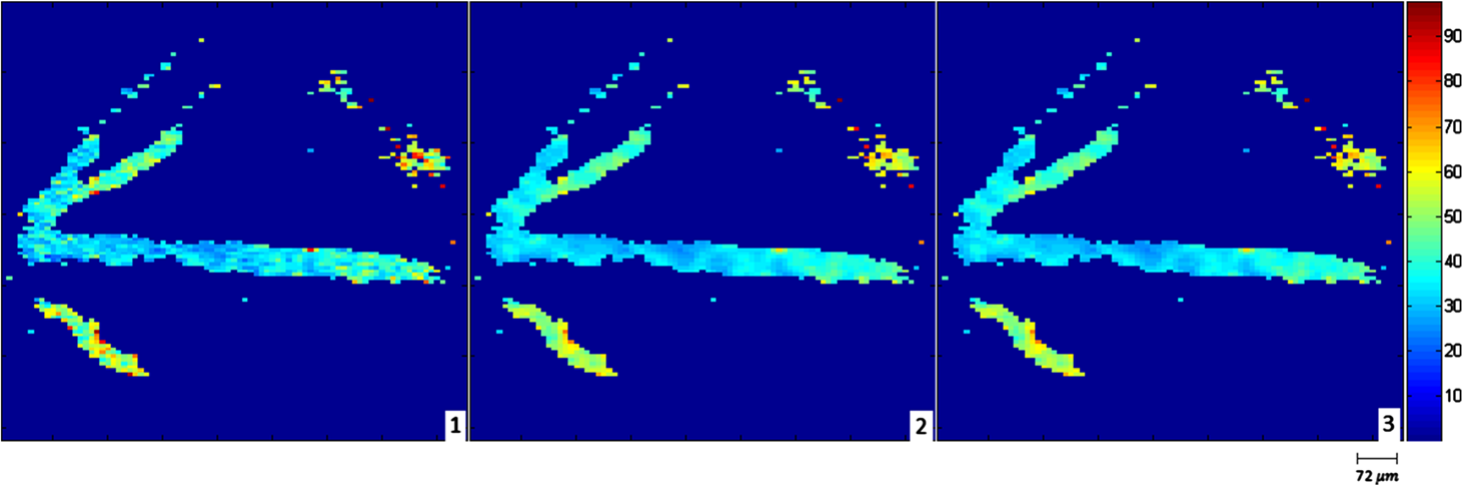
**The rectangular frame in Figure **1**(1) and its estimates in the presence of noise with 20 dB SNR using the LS (2) and proposed RLS (3) methods.** The color bar shows oxygen tension in millimeters of mercury.

Figure 8 compares the visual results of the proposed and LS estimation methods for the frame shown in the rectangle in Figure 1. This illustrates the artifacts of the LS estimation more clearly. As mentioned in [14], the RLS estimation generates smoother *pO*_2_ maps whose values fall within the physiologically expected range. The acquisition of the real oxygen tension map of a rat’s retina, shown in Figure 3, is described in [6]. In Figure 3, we compare the visual results of the proposed RLS, iterative RLS, and LS estimation methods for the real data. When compared with the LS result, the iterative and proposed RLS methods generate smoother oxygen tension maps.

## Discussions and conclusion

Presently, by use of PLIM method, available oxygen tension maps have frame sizes around 512x160 pixels. As the imaging instruments become enhanced, this size will rise considerably. The RLS method implemented in an iterative way can handle oxygen tension images having this size of frames but as shown in the Simulated and Experimental Data Results Section, it becomes slower as size of the images gets larger. The proposed method addresses this problem and can be used even for much larger oxygen tension maps. On the other hand, the proposed method is limited by the assumption made when developing the regularization window that variation among oxygen tension values of retinal vessels within this size of window should be minimal. For the problems in which this assumption is not valid, the proposed method can generate unsatisfactory results. In this study, by exploiting intrinsic properties of the problem, we have developed a fast RLS estimation method that is applicable to large oxygen tension maps. By comparing the results of the available and proposed approaches, it was shown that, although there is a minor difference in estimation performance, the proposed method is significantly faster. Additionally, the proposed method is not restricted to the problem of oxygen tension estimation, and is applicable to other problems where a similarly strong relationship exists between neighboring pixels.

## Abbreviations

LS: Least squares; RLS: Regularized least squares; PLIM: Phosphorescence lifetime imaging; FLIM: Fluorescence lifetime imaging; pO2: Oxygen tension.

## Competing interests

The authors declare that they have no competing interests.

## Authors’ contributions

Each Author has contributed substantially to the preparation of the paper and approves of its submission to the Journal. Both authors read and approved the final manuscript.
